# Chemical, Biochemical, and Microbiological Properties of Soils from Abandoned and Extensively Cultivated Olive Orchards

**DOI:** 10.1155/2013/496278

**Published:** 2013-11-17

**Authors:** A. M. Palese, R. Magno, T. Casacchia, M. Curci, S. Baronti, F. Miglietta, C. Crecchio, C. Xiloyannis, A. Sofo

**Affiliations:** ^1^Dipartimento delle Culture Europee e del Mediterraneo: Architettura, Ambiente, Patrimoni Culturali (DiCEM), Università degli Studi della Basilicata, Via San Rocco 3, 75100 Matera, Italy; ^2^IBIMET-CNR, Istituto di Biometeorologia, Via G. Caproni 8, 50145 Firenze, Italy; ^3^CRA, Centro di Ricerca per l'Olivicoltura e l'Industria Olearia, C. da Li Rocchi-Vermicelli, 87036 Rende, Italy; ^4^Dipartimento di Biologia e Chimica Agroforestale e Ambientale, Università degli Studi di Bari, Via Orabona 4, 70126 Bari, Italy; ^5^Scuola di Scienze Agrarie, Forestali, Alimentari ed Ambientali, Università degli Studi della Basilicata, Viale dell'Ateneo Lucano 10, 85100 Potenza, Italy

## Abstract

The abandonment of olive orchards is a phenomenon of great importance triggered mainly by economic and social causes. The aim of this study was to investigate some chemical, biochemical, and microbiological properties in a soil of a southern olive grove abandoned for 25 years. In order to define the effect of the long-term land abandonment on soil properties, an adjacent olive grove managed according to extensive practices was taken as reference (essentially minimum tillage and no fertilization). Soil organic matter, total nitrogen, and pH were significantly higher in the abandoned olive grove due to the absence of tillage and the natural inputs of organic matter at high C/N ratio which, *inter alia*, increased the number of cellulolytic bacteria and stimulated the activity of **β**-glucosidase, an indicator of a more advanced stage of soil evolution. The soil of the abandoned olive orchard showed a lower number of total bacteria and fungi and a lower microbial diversity, measured by means of the Biolog method, as a result of a sort of specialization trend towards low quality organic substrates. From this point of view, the extensive cultivation management seemed to not induce a disturbance to microbiological communities.

## 1. Introduction

Olive trees have been cultivated for centuries becoming one of the most representative and stable crops, with about 9.5 million of hectares [1], and an integral part of the Mediterranean landscape, especially in the hilly and marginal parts of this basin [2]. In spite of this, the low yields compared with the high costs of the mechanical operations (harvesting and pruning), the unfavourable oil price, the competition with the foreign productions, and the reduction of the European Community financial aids, determined in the last decades a progressive olive orchard abandonment, particularly in marginal and inland agricultural areas of European producing countries. The consequence of this phenomenon can be a positive restoration of natural vegetation or a negative soil degradation due to the absence of conservation practices, especially on slope lands [3, 4].

In an abandoned olive orchard trees assume the original bushy form, canopies become dense and closed, and pioneer vegetation recolonizes free spaces according to ecological successions that tend to arrive, after long time, to a natural formation (“climax”) where soil and vegetation components are *in equilibrium* [2, 5, 6]. During the transition of an olive grove from a “disturbed” (cultivated) condition to a climax phase, we assume that soil properties progressively change, even if very slowly, as found in other similar agricultural systems [7, 8].

Several studies were conducted on the effect of abandonment of marginal agriculture in the Mediterranean area, most of them focused on the variability of physical and chemical properties of soils during progressive vegetation cover changes [9–11], but few field researches include biochemical and microbiological factors as indicators of soil fertility restoration, especially for olive orchards [7, 8, 12–16]. Therefore, the present study aimed to investigate some chemical, biochemical, and microbiological soil parameters of an olive orchard abandoned for 25 years. In order to evaluate the effect of the long-term land abandonment on soil quality, measurements were also carried out in a contiguous traditional olive orchard cultivated according to extensive agricultural practices, that is, low inputs of labour and materials [17].

## 2. Materials and Methods

### 2.1. The Experimental Olive Orchard

The study was carried out in a 2 ha rainfed olive orchard located on a flat land in the rural area of Lucera (south-eastern Italy, Puglia region; 41°27′38.32′′N, 15°22′13.75′′E). Soil is a Vertisol, classified as a Typic Calcixererts, fine, mixed, thermic, sandy clay loam (USDA soil taxonomy). Climate is typically Mediterranean, characterized by hot and dry summer, with a mean annual temperature of 15.7°C and an average rainfall of 583 mm year^−1^.

Olive trees (*Olea europaea* L., cv. Perenzana) were planted in 1970. In 1985, 3/4 of the orchard were completely abandoned, and its appearance has taken the form of Mediterranean coppice with shrubs, herbs, and weeds colonizing the space between trees and rows (“abandoned” olive grove). In the “managed” olive orchard, pruning was made every two years and residues were burned far from the field. Shallow tillage (15-cm depth) was carried out two times per year at the beginning of Spring and in Autumn before harvest in order to bury weeds and grasses. During the interval between such dates the soil remained covered by the spontaneous vegetation. No fertilizer application was performed. The trees in the managed part of the field (220 trees ha^−1^) were constituted from a single trunk (73%) or from two trunks (26%), while in the abandoned part the main trunk was difficult to identify, as suckers were predominant (up to 11). 

### 2.2. Soil Sampling Strategy and Chemical Analyses

In July 2010, three composite soil samples (each obtained on site by four pooled cores of seven cm of diameter) were collected from the top soil layers (0–20 and 20–40 cm) along the interrows of both managed and abandoned olive orchards. Samples were stored at 4°C in sterile plastic bags until the following analyses. 

Soil subsamples were air dried and sieved at 2 mm. On these samples the following analyses were carried out according to the official methods of soil analysis [18]: electrical conductivity and pH, measured on a mixture of soil (10 g dry weight) and distilled water (25 mL) and shaken for at least 2 hours on a shaker at 40 rpm; soil organic matter (SOM) by the dichromate oxidation method; total nitrogen by the Kjeldahl method; available phosphorus by the Olsen method.

Soil enzyme activities were measured on fresh soil samples. *β*-glucosidase activity was determined according to Eivazi and Tabatabai [19] and expressed as mg *p*-nitrophenol h^−1^ g^−1^ soil. The dehydrogenase assay was performed according to the method of Von Mercì and Schinner [20] and the activity expressed as mg triphenylformazan h^−1^ g^−1^ soil. For cellulase, measured by the method of Hope and Burns [21], the activity was expressed as g glucose h^−1^ g^−1^ soil. The enzyme activities were expressed as units per g of dry soil. 

### 2.3. Soil Microbial Counts and Microbial Community Metabolic Profiles (Biolog)

Three replicates of 5 g subsamples (dry weight equivalent) of each fresh soil sample were suspended in 45 mL sterile 0.1% sodium pyrophosphate-one quarter strength Ringer solution and sonicated at 35 kHz for 2 min to disperse microbial cells. Tenfold serial dilutions of the supernatants were made in a sterile Ringer solution. Aliquots were spread plated in triplicate on 1/10 strength Tryptic Soy Agar (TSA) medium amended with cycloheximide 0.1 mg mL^−1^ for bacterial counting, and inoculated in Malt Extract Agar (MEA) medium implemented with streptomycin 0.03 mg mL^−1^ and tetracycline 0.02 mg mL^−1^[22] in triplicate for fungal counting. Cellulolytic bacteria were spread and included between two double agar layers, using the following culture medium: cellulose powder 5 g L^−1^, NH_4_H_2_PO_4_ 2 g L^−1^, KH_2_PO_4_ 0.6 g L^−1^, K_2_HPO_4_ g L^−1^, MgSO_4_·7 H_2_O 0.8 g L^−1^, tiamine 0.1 mg L^−1^, adenine 4 mg L^−1^, adenosine mg L^−1^, yeast extract 0.5 g L^−1^, and agar 17 g L^−1^. Counting took place after suitable incubation period (72 h for bacteria and 120 h for fungi) at 28°C.

Sole carbon source utilization patterns of soil microbial communities, also called community-level physiological profiles (CLPPs), were assessed using the Biolog 96-well Eco-Microplates (AES Laboratoire, France), containing 31 different carbon sources, three times replicated. Data were analysed to determine metabolic diversity indices, including Average Well Colour Development (AWCD) (the mean of the blanked absorbance values for all the substrates), Shannon's substrate diversity index (*H*′), substrate evenness (*E*, equitability of activities across all utilized substrates), and substrate richness (*S*, the number of utilized substrates), according to Zak et al. [23] and Sofo et al. [24]. The microplates were incubated at 25°C in the dark and colour development was measured as optical density (OD) every 24 h over a 144 h period using a Microplate E-Max Reader (Bio-Rad) with a E590 nm wavelength filter. The substrate utilization profiles were analyzed on well absorbance values at the 96 h observation period.

### 2.4. Statistical Analysis

The statistical analysis of data was carried out using the SigmaStat 3.1 SPSS Inc. software. Two-way analysis of variance (ANOVA) of the soil parameters was performed with orchard management and soil depth as fixed factors. Means were separated according to Duncan's multiple comparison test at *P* < 0.05 and *P* < 0.01. Relationships among soil properties were studied using Pearson correlations. The number of measured samples is specified throughout the text and in the figure captions.

## 3. Results and Discussion

Soil electrical conductivity did not differ between the two systems, whereas pH showed significantly higher values in the managed orchard (Table 1). The lowering of pH in the abandoned grove could be attributed to the quality of the organic material, particularly rich in soil-acidifying compounds, such as polyphenols and organic acids contained in olive leaves and fruits [25].

SOM is a fertility parameter that responds to changes in soil management in the long term [26]. SOM of the abandoned olive orchard was significantly higher than that found in the managed treatment (Table 1). This increase was related to both the lack of soil disturbance by tillage [27] and the continuous natural inputs of organic matter occurring during 25 years of abandonment which provided the soil of carbon and energy sources [13]. These inputs were derived from olive trees and shrub-herbaceous plants (olive fruits, senescent leaves, shoots, and branches; other plant aboveground biomass; roots; root exudates), which settled widely in the free spaces between the interrow areas, and produced a low quality litter characterized by high content of lignin and polyphenols or a low content in N (C/N > 25) [28]. In any case, even the soil of the managed olive showed a good level of SOM due to both its pedologic origin (Vertisol: a deep black clay soil) [29] and to soil extensive management (minimum tillage and weed burial). Similarly, Álvarez et al. [3] found that Soil Organic Carbon (SOC) content of organic olive groves (tilled once a year usually in spring or grazed at different intensities), located in Southern Spain, was relatively high compared with the values reported for rainfed agricultural soils in the region (below 1%). The authors also found that SOC contents tended to be higher in undisturbed areas with natural vegetation than in the abovementioned organic olive orchards.

The measured total nitrogen in the abandoned grove (1.9 g kg^−1^ in the 0–40 cm soil layer) was higher (*P* < 0.001) than the value observed in the managed system (1.3 g kg^−1^) (Table 1). Furthermore, a good correlation was found between organic matter and total nitrogen (*r* = 0.91; *P* < 0.001). 

No significant differences between the two management systems were found in the C/N ratio, which fluctuated between 11.8 and 12.2 in the 0–40 cm soil layer (Table 1). A soil C/N ratio of 10 is considered optimal for the best incorporation rate of the organic matter into the soil profile [10]. 

The content of *P*_Olsen_ in soil of managed olive orchard was significantly higher than in the abandoned treatment (*P* < 0.001) (Table 1). Probably, the phosphorus in plant residues, which were buried three months before soil sampling (July 2010), became available during their decomposition. Nevertheless, *P*_Olsen_ in the abandoned soil was enough to support vegetation growth and development [3].

Total bacteria and fungi were more numerous in the managed orchard than in the abandoned one (Table 2).

However, the importance of these groups of microorganisms in terms of organic matter decomposition and other biochemical changes is linked to their relative abundance and functional diversity due to the metabolism and not to their numbers [30]. In the present study, the bacteria to fungi ratio was higher in soil of the abandoned orchard (1.6 versus 1.2 in the 0–40 cm soil layer), indicating that in this treatment bacterial decomposition was favored over fungal decomposition. For cellulolytic bacteria, an opposite trend was found being higher in the abandoned grove (Table 2). This could be probably due to the high inputs of cellulosic material deriving from a more dense shrub vegetation occurring in the abandoned olive grove. It is well known that plant species (in terms of quantity and quality) strongly affect the composition of microbial communities during vegetation succession by means of rhizodeposition and the decay of litter and roots [13, 31]. The change in soil energy pathways could determine, as a consequence, a taxonomic shift in the composition, activity, and functional diversity of soil microbial biomass [32]. 

The extracellular soil enzyme *β*-glucosidase hydrolyzes organic matter, so releasing glycosidic residues such as glucose and galactose. The activity of this enzyme is an excellent indicator of the degree of evolution and maturity of a soil. It increases in the final stages of an ecological succession as it is related to biomass turnover and strongly depends on the soil management adopted [14, 19, 33, 34]. In this study, *β*-glucosidase activity was significantly higher in the abandoned orchard (Table 2), which resembles more the later stages of an ecological succession (climax), and was significantly related to SOM (*r* = 0.88; *P* < 0.001). In contrast, dehydrogenases isoforms are common to most organisms, with a predominantly intracellular localization. They are good indicators of the viability of bacterial populations and their oxidative metabolism [20]. In our case, dehydrogenase activity did not differ statistically between the two treatments and appeared to be strongly influenced by soil depth (Table 2). Finally, cellulases are a family of enzymes, mainly produced by fungi, bacteria, and protozoa, belonging to the family of hydrolases, which catalyze the hydrolysis of 1,4-*β*-D glycosidic bonds of cellulose. Their activity was not different from a statistical point of view (Table 2).

The functional diversity of soil microbial communities estimated by the Biolog metabolic assay is based on the ability of the microbial strains to oxidize different carbon sources and it has a high discriminating power among microbial soil communities [23]. The community-level physiological profile (CLPP) obtained by this method was used to differentiate the soil bacterial populations of the two orchards. Data show that, among the indexes of microbial diversity examined, S and AWCD were significantly higher in the managed system (Table 3) indicating a higher bacterial functional diversity and complexity of this part of the grove.

**Figure 1:**
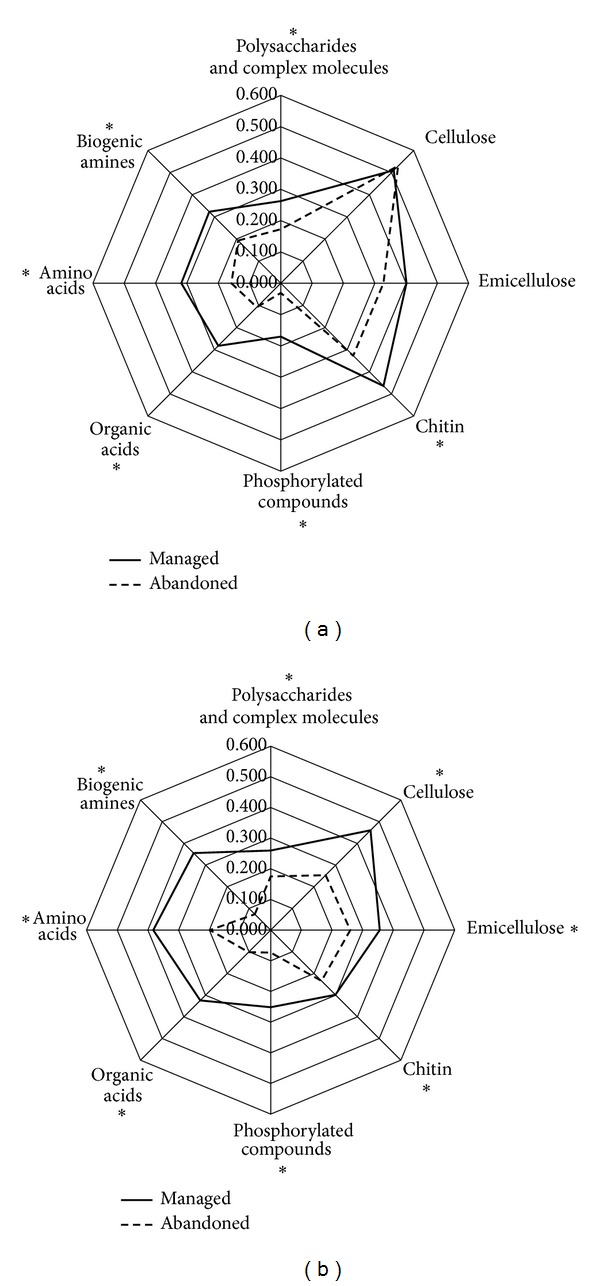
Radar diagrams of the average well colour development (AWCD) values for the eight main groups of the Biolog carbon compounds in the 0–20 cm (a) and 20–40 cm (b) soil layer of managed (continuous line) and abandoned orchard (dashed line). The means (*n* = 9) with the asterisk are significantly different between the two systems at *P* < 0.01.

The 31 carbon substrates of the Biolog plates can be divided into eight main groups (polysaccharides and complex molecules, cellulose, hemicellulose, chitin, phosphorylated compounds, organic acids, aminoacids, and biogenic amines). The radar diagrams of AWCD values of these groups are reported in Figure 1. As AWCD values provide a measure of the cultural bacterial activity for each group of compounds, it is noteworthy that, with the exception of cellulose and hemicellulose in the 0–20 cm soil layer, bacterial activity due to substrate degradation was significantly higher in the managed orchard. This is in accordance with the differences found in total bacteria and total AWCD which were significantly higher in the managed soil than in the abandoned one (Tables 1 and 3). Interestingly, in the surface soil layer cellulose and emicellulose bacterial degradation did not show significant differences (Figure 1) and explain the differences in cellulose activity between cultivated and abandoned orchards reported in Table 2.

The higher bacterial functional diversity and complexity recorded in the managed orchard could be due to the more biodegradable substrates which were highly palatable to microbial communities. In fact, simple organic carbon such as simple sugars and amino acids are quickly absorbed and provide nutrients to microbes [35]. Probably, soil biota of the cultivated orchard were positively affected by the high litter quality (in terms of less phenolic substances and more nitrogen) produced within the managed olive orchard (essentially plants belonging to Gramineae, Composite, and Leguminosae families), periodically buried into the soil by tillage and thus more easily decomposable. By contrast, the increase of low quality organic inputs in the abandoned soil seemed to determine a bacterial specialization towards substrates characterized by high C/N ratio and low degradation rate resulting in a fewer ecological niches. 

## 4. Conclusions

Chemical changes occurred in the soil of the olive orchard after 25 years from the abandonment were evident and to some extent predictable due to the natural inputs of organic matter and the absence of tillage. Likewise, the high *β*-glucosidase activity, strongly related to soil organic matter, placed soil from abandoned grove in an advanced evolutionary stage. On the other hand, the study of the carbon substrate utilization profiles, performed by the Biolog method, highlighted unexpectedly a lower microbial diversity in the abandoned orchard. Taking into account this finding, it would be advisable to deepen such aspect by studying soil development in abandoned olive orchards using a chronosequence approach.

## Figures and Tables

**Table 1:** Two-way ANOVA analysis of chemical properties of the soils studied (average values; *n* = 6).

Treatment	SOM (g kg^−1^)	Total N (g kg^−1^)	C/N	Available *P* (mg kg^−1^)	pH	Electric conductivity (*μ*S cm^−1^)
Management	*P* = 0.016	*P* < 0.0001	ns	*P* < 0.0001	*P* = 0.0002	ns
Managed	27.7 b	1.3 b	12.2 a	56.0 a	7.9 a	80.4 a
Abandoned	38.6 a	1.9 a	11.8 a	31.5 b	7.1 b	70.0 a
Soil depth	ns	ns	ns	ns	*P* = 0.0085	ns
0–20 cm	33.2 a	1.6 a	12.0 a	43.7 a	7.5 a	80.3 a
20–40 cm	33.1 a	1.6 a	12.0 a	41.4 a	7.4 b	70.1 a
Management × soil depth	ns	ns	ns	ns	*P* = 0.0017	ns

ns: not significant; mean values followed by different letters are significantly different between the two management systems.

**Table 2:** Two-way ANOVA analysis of microbial counts and enzyme activities of the soils studied (average values; *n* = 6).

Treatment	Total bacteria (log CFU g^−1^)	Total fungi (log CFU g^−1^)	Cellulolytic bacteria (log CFU g^−1^)	*β*-glucosidase (units g^−1^)	Dehydrogenase (units g^−1^)	Cellulase (units g^−1^)
Management	*P* = 0.0004	*P* < 0.0001	*P* < 0.0001	*P* = 0.0004	ns	ns
Managed	9.4 a	8.0 a	2.4 b	16.5 b	89.3 a	13.6 a
Abandoned	8.7 b	5.6 b	4.6 a	36.5 a	92.5 a	16.0 a
Soil depth	*P* = 0.0038	ns	ns	ns	*P* = 0.0176	ns
0–20 cm	8.8 b	6.9 a	3.5 a	25.6 a	105.7 a	14.7 a
20–40 cm	9.3 a	6.8 a	3.5 a	27.5 a	76.0 b	14.8 a
Management × soil depth	*P* = 0.0007	*P* = 0.0032	*P* = 0.0023	ns	ns	ns

ns: not significant; mean values followed by different letters are significantly different between the two management systems.

**Table 3:** Two-way ANOVA analysis of the indices used for the Community Level Physiological Profiling (CLPP; Biolog method) in the studied soils (average values; *n* = 9).

Treatment	H′	S	E	AWCD
Management	ns	*P* = 0.0003	ns	*P* = 0.0047
Managed	2.5 a	12.3 a	2.4 a	0.33 a
Abandoned	2.1 a	8.6 b	2.2 a	0.19 b
Soil depth	ns	ns	ns	ns
0–20 cm	2.4 a	10.6 a	2.4 a	0.27 a
20–40 cm	2.3 a	10.5 a	2.3 a	0.25 a
Management × soil depth	ns	ns	ns	ns

ns: not significant; mean values followed by different letters are significantly different between the two management systems. H′: Shannon's substrate diversity index; S: substrate richness; E: substrate evenness; AWCD: average well colour development.
